# Etching Patterns of Self-Etching Primers in Relation to Shear Bond Strength on Unground Enamel Samples

**DOI:** 10.3390/dj9110138

**Published:** 2021-11-20

**Authors:** Lorenz Brauchli, Markus Steineck

**Affiliations:** Department of Orthodontics, School of Dentistry, University of Basel, 4051 Basel, Switzerland; Markus.Steineck@unibas.ch

**Keywords:** dentistry, composite, self-etching primer, etching pattern, shear bond strength

## Abstract

It was the intention of the study to evaluate the etching effects of several self-etching primers on unground enamel and their relevance for shear bond strength testing. Seven self-etching primers (Clearfil SE, Futurabond NR, M-Bond, One Coat, Optibond, Transbond SEP+, Xeno III) and a conventional 35% phosphoric gel acid were applied to bovine incisors according to the manufacturer’s instructions. All specimens were analyzed by electron microscopy. A visual four-step grading was used for the characterization of the macroscopic (5000×) and microscopic (20,000×) etching patterns. In addition, shear bond strength for all the products was tested with an Instron 3344 after 1000 thermocycles between 5 °C and 55 °C. Statistical analysis was carried out using Kruskal–Wallis with Dunn’s post-test and Pearson’s correlation coefficient. Very strong etching patterns with well-defined prisms were found for the conventional etching, Transbond SEP+, and to a lesser degree, for Xeno III. Clearfil SE and Futurabond NR revealed moderate etching patterns, and M-Bond, One Coat, and Optibond revealed very weak etching patterns. The bond strength correlated well with the etching patterns. The highest shear strength was obtained with conventional etching and Transbond SEP+, followed by Clearfil SE. Moderate shear bond strengths were found for Xeno III, Futurabond NR, One Coat, and M-Bond, and the lowest were found with Optibond.

## 1. Introduction

Since its introduction in orthodontic practice by Newman in 1965 [1], adhesive technologies today are essential in orthodontic therapy. Since the mechanical interlocking of the bonding monomers to the enamel surface plays a major role in determining adhesive strength [2,3,4], etching is a crucial stage in the bonding procedure. It has been documented that conventional etching with a 35% phosphoric acid for 15–60 s leads to a well-defined etching pattern with dissolution of interprismatic material [5]. A roughened and porous surface is produced with alteration of the enamel [6] to a depth of up to 200 µ and a fine microstructure, which considerably increases the enamel surface area [3]. Self-etching primers on the other hand react differently. They dissolve the enamel, and simultaneously, the monomer penetrates the retentive relief to the same depth. At the same time, the etching is buffered by the dissolved substrate. This method is a simplified technique with a reduced potential for contamination. The etching pattern created by self-etching primers is generally reported to be less distinctive than for conventional etching [7]. However, there is no consensus on the suitability of self-etching primers for the use on unground enamel. Whereas some authors have reported similar bond strength to conventional etching [8,9,10], others found lower bond strengths [11,12].

It was the aim of the present study to evaluate the etching pattern following the application of several different self-etching primers, according to the manufacturer’s instructions, and to investigate the bond strengths as measured by shear bond strength testing.

## 2. Materials and Methods

Etching pattern: 32 freshly extracted bovine incisors were used as substitute for human enamel and divided into 8 groups of four incisors each. Sample size was arbitrarily chosen. Seven self-etching primers (Transbond SEP(3M/unitek, Monrovia, CA, USA), Xeno III (Dentsply, Constance, Germany), Clearfil SE (Kuraray, Noritake Dental, Tokyo, Japan), Futurabond NR (Voco, Cuxhaven, Germany), One Coat (Coltene/Whaledent, Cuyahoga Falls, OH, USA), M-Bond (Tokuyama, Burlingame, CA, USA), and Opti-Bond (Kerr GmbH, Bioggio, Switzerland)) and a conventional 35% phosphoric acid gel (Transbond XT Etching Gel, 35% phosphoric acid, 3M/Unitek, Monrovia, CA, USA) were applied to the bovine incisors, according to the manufacturer’s instructions. An application time of 20 s was recommended for most products (Clearfil SE, Futurabond NR, One Coat, Xeno III), although Transbond SEP+ was applied for 10 s, Optibond for 2 × 20 s, and M-Bond for 30 s. The conventional etching was applied for 30 s. All specimens were thoroughly rinsed with water using the chairside syringe to eliminate residue from the primer. The specimens were sputtered with gold (Sputter Coater SCD 005, Baltek Corp., Northvale, NJ, USA), and two locations from each of the four teeth in each group were observed under an electron scanning microscope at a magnification of 5000 and 20,000 times at 30.0 kV acceleration voltage (ESEM, Philips 30, Royal Philips Electronics, The Netherlands), resulting in a total of 16 measurements for each primer.

**Figure 1:**
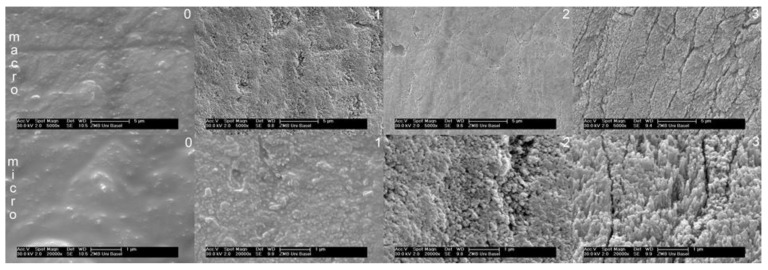
SEM images representing the visual four point grading system. Upper row: macroscopic grades 0–3. Lower row: microscopic grades 0–3.

A grading system at four levels was defined for the macroscopic and microscopic etching patterns (Figure 1): Macrostructure: 0 = minimal or no change, 1 = no defined prisms but well defined roughening, 2 = prisms apparent but poorly defined, 3 = well defined prisms. Microstructure: 0 = smooth surface, 1 = superficial roughness, 2 = granular surface, low etching depth, 3 = filiform surface, high etching depth.

All SEM images were twice classified by one blinded examiner with an interval of two weeks. The results were statistically analyzed for mean and standard deviations. In addition, a Kruskal–Wallis with Dunn’s multiple comparisons post-test was applied to the data for ranking the etching patterns (Prism 5.04/d, GraphPad, San Diego, CA, USA). The level of significance was set at *p* ≤ 0.05. The relevance of the macroscopic and microscopic etching pattern to the shear bond strength was calculated by the Pearson’s correlation coefficient.

Shear bond strength: 20 bovine incisors for each group were conditioned with the seven self-etching primers and the conventional phosphoric acid, as mentioned above. Sample size was arbitrarily chosen. Composite cylinders were used as shear bodies, which were produced with a silicon replica of a highly precise CNC milled stainless steel form (Picomax 60-M/HSC, Fehlmann AG, Seon, Switzerland). Grandio Flow (VoCo, Cuxhaven, Germany) was used to fill the cylinders. Grandio Flow is a HEDMA/BISGMA-based 80% filled nanohybrid composite. The surface of the flat front side of the cylinder was 12.6 mm^2^. The cylinders were bonded to the teeth with Transbond XT (3M/Unitek, Monrovia, CA, USA). Transbond XT is a BISGMA-based and 80% quartz and silica filled non-flowable composite. In the case of the conventional etching, Transbond MIP (3M/Unitek, Monrovia, California, USA) was used as primer. All specimens were subjected to 1000 thermocycles between 5 °C and 55 °C. The teeth were embedded into polymethacrylate sockets (Technovit, Heraeus Kulzer, Wehrheim, Germany), with the bonded enamel surface parallel to the shear force vector. Maximum shear bond strengths were recorded with an Instron 3344 (Instron Corp., Wilmington, DE, USA). Statistically, the data were analyzed as mentioned above. Normality of distribution was not calculated, since a nonparametric method was used.

pH: Finally, pH measurements were taken using indicator strips with a 0.5 step pH scale (Acilit pH 0-6, Merck KGaA, Darmstadt, Germany). The strips were tested with four buffer solutions of different pH. If the self-etching primer could not be matched exactly to one color scale, the mean between the two closest scales was taken.

## 3. Results

**Figure 2:**
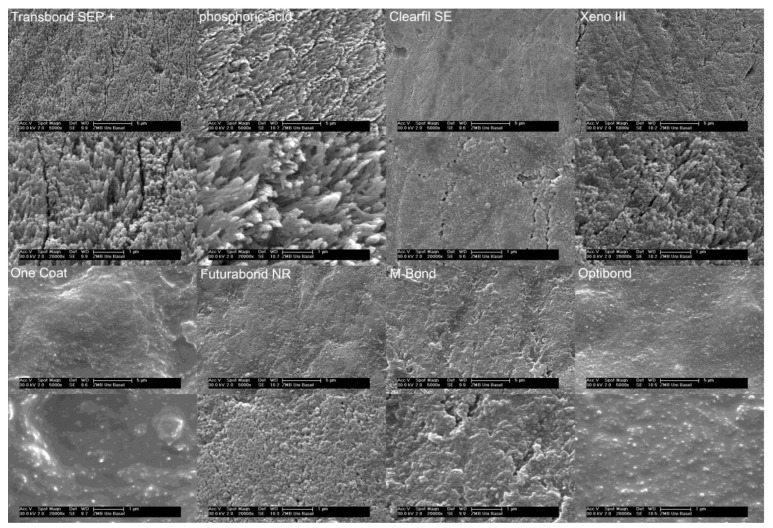
Representative SEM images of all investigated products. Macroscopic etching patterns are shown with the product name. The corresponding microscopic etching patterns of each product are shown right below the macroscopic SEM images.

Etching patterns: The strongest etching patterns were observed after conventional etching and application of Transbond SEP+. A significant difference (*p* ≤ 0.05) was found in both the macro- and micro-retentive etching patterns of the enamel surface after application of M-Bond, One Coat, and Opti-Bond in comparison with the previous two etchants. The etching patterns for these three products showed only minimal roughening (Figure 2, Table 1). The squared correlation coefficient r^2^ for the relationship between macroscopic and microscopic etching patterns was 0.92.

Shear bond strength: The highest bonding forces were observed with Transbond SEP+ (34.2 MPA) and conventional etching (32.3 MPa). The lowest forces were found for One Coat (11.5 MPa), Futurabond NR (9 MPa), and M-Bond (8.6 MPa). Opti-Bond was not evaluated, as all bonding interfaces failed during the thermocycles (Table 1). The squared correlation factors r^2^ between the macro-retentive surface and the shear bond strength was 0.78 and between micro-retentive enamel surface and the shear bond strength, 0.58.

pH: The squared correlation coefficient r^2^ for the relationship between macroscopic and microscopic etching patterns and pH was −0.86 and −0.92, respectively. The correlation between pH and shear bond strength was −0.52. pH values are shown in Table 1.

## 4. Discussion

Due to restricted access to the SEM laboratory, only four incisors per self-etching adhesive could be evaluated in this investigation. Although the etching patterns were fairly consistent among the samples of each group, this was clearly limiting the power of the macro-retentive and micro-retentive grading as well as its correlation to the shear bond strength of the respective products. Another limitation might be the use of bovine incisors instead of human teeth. However, the use of bovine enamel instead of human enamel has been recommended by the ISO 11,405 norm for adhesive shear testing and is well documented in the literature where similar [13,14,15,16] or slightly reduced [17] bond strengths were found. The retentive etching pattern was found to differ only slightly between the two species with no effect on bond strength [15]. Histochemical as well as anatomical observations for both species were found to be essentially similar [15,16,18]. The use of bovine instead of human enamel seems, therefore, to be justified but must be considered as a further limitation.

The grading system used is derived from the one proposed by Hobson [19]. The major modification is the use of a digital four-point scale, which can be used for statistical calculations and the development of an additional microscopic four-point scale.

The etching patterns observed for the conventional phosphoric acid are in good agreement with those reported in the literature for human and bovine substrates [9,10,20], but the etching patterns were less well expressed than those observed by Cal-Neto [21] on human premolars. The patterns observed for the self-etching primers showed a large variation. Whereas, Transbond SEP+ showed similar readings on both the macroscopic and microscopic scales as the phosphoric acid; others did not. In particular, products with a pH > 3 (One Coat, Opti-Bond) did not achieve a clearly retentive pattern, although it still exceeds the critical pH value [22] for decalcification of hydroxyapatite (pH 5.5) or fluoroapathite (pH 4.5). The quality of the etching pattern was inversely related to the pH of the self-etching primer. This relation was very close with r^2^ values around −0.9 and in agreement with the current literature [9,20]. Therefore, a prolonged application time might be beneficial for self-etching primers with a higher pH. In addition, a low pH resulted not only in a more pronounced but also in a more uniform etching pattern. It seems that the intact enamel surface has an irregular resistance to acid etching, which becomes irrelevant if strong etchants are applied. Irregular etching patterns within a single tooth surface have also been described in previous investigations [8,23]. They might be due to areas of aprismatic enamel [24]. The quality of the macro-retentive as well as the micro-retentive etching patterns were found to be closely related with an r^2^ of 0.92. Therefore, highly macro-retentive etching patterns with clearly identifiable enamel prisms also showed the most filigree microstructures, probably leading to the largest surface areas.

For testing of shear bond strength, 1000 thermocycles between 5 °C and 55 °C with a transfer time of 2 s were used. This is significantly less than the 10,000 cycles proposed in a previous study [25]. However, in contrast to restorative dentistry, the duration of the orthodontic bond is per se limited, since the appliances are designed to be removed at the end of treatment. Thus, a reduced amount of cycles seemed appropriate.

The shear bond strength measured was correlated better with macro-retentive (r^2^ = 0.78) rather than with micro-retentive (r^2^ = 0.58) patterns or pH (r^2^ = 0.52). This was surprising, as it is suggested that microstructures might have more of an influence on shear bond strength [2,3,8,26]. However, the difference found in the present study is relatively small, and when considering the ranking according to the Kruskal–Wallis post-test, a better correlation is found for the micro-retentive structures. The relation of shear bond strength to etching patterns is in general agreement with many previous studies [8,9,12,20,23,27]. However, interestingly, no significant difference for shear bond strength was found between Transbond SEP+ and Clearfil SE, although they display distinctively different etching patterns. Probably, other factors such as resin composition play an important role as well. In the case of Clearfil SE, the addition of MDP, which is known for its strong adhesive qualities [28], might compensate for the less retentive etching pattern. Additionally, some unexplored variables can have a significant influence on the oral environment and can significantly affect bond strength. The use of probiotics [29] and natural compounds [30] can modify clinical and microbiological parameters and could, thus, have an effect also for the variables tested in the present report. These concerns should be considered in future laboratory research and clinical trials.

Clinically, the etching pattern and shear bond strengths are only of interest if they are linked to a prolonged bond survival. This relationship seems to be complex. Apart from the higher occlusal forces on the posterior teeth, the higher bracket failure rate in the posterior occlusion might be due to more aprismatic enamel [27] and less defined etching patterns [19]. It is interesting that in vivo, the etching quality was closely related to bond survival, whereas in vitro, the bond strength of self-etching primers with a reduced etching pattern is often not significantly different from conventionally etched samples [8,9,10]. Finally, it has to be remembered that aggressive etchants lead to a more pronounced loss of enamel. Therefore, the combination of a strongly adhesive resin and a moderately strong etchant such as Clearfil SE would seem to be desirable.

## 5. Conclusions

Macro-retentive as well as micro-retentive etching patterns are closely related to the pH of the etchant as well as to the bond strength in shear testing. Whereas Transbond SEP+ achieved the highest bond strengths of all the self-etching primers, Clearfil SE seems very promising in combining a moderate etching pattern with a high bond strength.

## Figures and Tables

**Table 1:** pH, shear bond strength, macro-/micro-etching patterns, and standard deviations. Significant differences between different products (*p* ≤ 0.05) are indicated by the group numbers.

	pH	Shear Bond Strength MPa (SD)	Significance	Macro-Etching Pattern	Significance *p* ≤ 0.05	Micro-Etching Pattern	Significance *p* ≤ 0.05
(1) H_3_PO_4_	0	32.2 (7.2)	3, 4, 5, 8	3 (0)	4, 5, 6	3 (0)	4, 5, 6
(2) Clearfil SE	2	26.8 (7.7)	3, 4, 5	1.8 (0.7)	-	2 (0)	-
(3) Futurabond NR	1.5	9 (4.2)	1, 2, 7	1.1 (0.4)	-	2.3 (0.5)	-
(4) M-Bond	1.5	8.6 (3.7)	1, 2, 7	0.88 (0.6)	1, 7, 8	1.6 (0.7)	1, 7, 8
(5) One Coat	3.25	11.5 (9.8)	1, 2, 7	0.13 (0.4)	1, 7	0.5 (0.8)	1, 7
(6) Optibond	3.25	-	-	0 (0)	1, 7	0.63 (0.5)	1, 7
(7) Transbond SEP+	0.5	34.2 (7.2)	3, 4, 5, 8	2.9 (0.4)	4, 5, 6	3 (0)	4, 5, 6
(8) Xeno III	1	15.2 (3.2)	1, 7	2.3 (0.7)	-	2.8 (0.5)	-

## Data Availability

Department of Orthodontics, University of Basel.
